# Thoughts on the origins, present, and future of the coronavirus crisis: marginalization, food and housing, and grassroots strategies

**DOI:** 10.1007/s10460-020-10064-2

**Published:** 2020-05-23

**Authors:** Antonio Roman-Alcalá

**Affiliations:** 1 Agroecology Research-Action Collective, San Francisco, USA; 2 Erasmus University, Rotterdam, The Netherlands

Few in the world of food system scholarship saw a pandemic crisis coming. Some did, like economic geographer Rob Wallace (2009), whose work has unraveled the social-ecological origins of novel pathogens in industrial agriculture and capitalist development. Such work has been generally underappreciated in food system scholarship, and even less appreciated in society at large. Only *after* a crisis appears do analyses like Wallace’s gain credence, and unfortunately, “the pathogen has left the barn”.

So here we are: a dysfunctional food system that works for capitalists, takes epidemiological risks, and operates on a baseline perpetuation of mass inequality, resulting in poorly nourished communities of poor people who suffer increased morbidity and mortality from diseases. Though inequality is not new, food studies have increasingly focused on social equity, analyzing food and farming in terms of their effects on the most marginalized (and mostly non-white) sectors in society: food workers, immigrants, low income people, small-scale and tenure-insecure farmers. Coronavirus simply brings existing inequalities into sharp relief.

It is inevitable that supply chain disruptions, changes in labor availability, and other coronavirus outcomes will shake up the current food system. Shortages, shifting marketing options for farmers, greater poverty and hunger, and return to home-scale subsistence strategies are likely outcomes. But it is very difficult to predict the details and trajectories of any of these particular developments.

Some food system scholars assume that breakdowns in the social order are likely to result in positive change. For instance, people are already repeating historic patterns of responding to crisis by planting home gardens and seeking local food options, in fear of supply breakdowns. Theoretically, this reduces reliance on the industrial model, and opens up spaces for alternatives to thrive. Yet we know from history that capitalist states intervene to preserve economic growth by bailing out highly-capitalized industries, and indeed the federal government has already allocated $23 billion to the agricultural sector. Meanwhile, gardening itself contains no inherent politics, with motivations from panic and patriotism to revolutionary antagonism. Movements must continue to actively shape *why* people garden, *how* they access land, and *who* can take part or benefit, if breakdowns are to result in positive transformation. They must also carefully consider their understanding of and approach to the state.

It is important to remember that the virus itself, and the people it moves through, and societal responses, will change and evolve over time. Social isolation is a real and current issue, and a fundamental shaker of established psychological and economic patterns, but it will not last forever. Those who want to prepare to make a better world out of this crisis should think through the next two years and beyond, in terms of various scenarios of isolation, government crackdown and increasing authoritarianism, and grassroots political responses from various sectors of society. Economic ramifications are key, considered as coevolving with shifts in values, politics, legitimacies, and emergent ‘from-below’ social effects. Preparation-focused thinking must consider food and agriculture, but cannot be limited to it, as narrow sectoral thinking and action prove themselves every day more inadequate.

For example, recent farmworker and food justice practitioner struggles for housing (rural and urban) indicate how housing access and affordability have *always* been problematic for working classes under capitalism. With coronavirus’s economic impacts, working people involved in food systems (and not) now face greater housing insecurity, and calls are growing for a ‘rent strike’. It is (past) time that people concerned with agriculture and human values also consider themselves concerned for housing and human values, and how these two issues are tied together and/or pitted against each other – and how they are linked fundamentally through capitalist relations of commodified land.

The hopeful news is that change strategies promoted for generations (often, especially, by the most marginalized) are still, if not more, relevant today. I speak here of slow, relationship-based, constructive, propositional, autonomous organizing efforts that reduce rather than increase reliance on problematic state politics. It seems foolish to expect effective bailouts from an uncaring kleptocratic state. Instead, strategies like solidarity food economies, unemployed associations, and community land trusted farmland and buildings can meet existing needs while linking food and housing, and advancing the visionary values and demands the moment requires.
